# Deciphering the best shape aspheric intraocular lens – A raytracing based optimisation study

**DOI:** 10.1016/j.zemedi.2025.07.004

**Published:** 2025-08-06

**Authors:** Achim Langenbucher, Jascha Wendelstein, Alan Cayless, Peter Hoffmann, Nóra Szentmáry

**Affiliations:** a Department of Experimental Ophthalmology, Saarland University, Homburg, Saar, Germany; b Department of Ophthalmology, Ludwig-Maximilian-University Clinics, Munich, Germany; c School of Physical Sciences, The Open University, Milton Keynes, United Kingdom; d Augen- und Laserklinik Castrop-Rauxel, Castrop-Rauxel, Germany; e Department of Ophthalmology, Semmelweis-University, Budapest, Hungary

**Keywords:** Best shape IOL, Raytracing, Nonlinear iterative optimisation, Spherical aberration correction, Best focus

## Abstract

**Purpose:**

To develop, implement and demonstrate a calculation strategy to derive the best shape spherical or aspherical intraocular lens (IOL) considering corneal spherical aberration (SA).

**Methods:**

The simulation concept is based on 2D raytracing and involves an ideal plano or spherical wavefront with an optical path length correction which simulates corneal SA. The IOL defined with its equivalent power PIOL, Coddington shape factor (CSF) and edge thickness (ET) could be located with its secondary principal plane (PP2) or its haptic plane (HP) at the predicted axial lens position (ELP).The lens geometry is optimised for the root-mean-squared wavefront error (RMSWF) and the best wavefront and rayscatter focus are derived.

**Results:**

The custom simulation software package is written in Matlab (version 2024a). The applicability of the simulation software is shown with some examples to show the performance of the results. The simulation results are structured to give some insight into best shape spherical and aspheric lenses, the impact of CSF, corneal spherical aberration to be corrected, and the concept of using the ELP to predict either the PP2 or the HP of the lens.

**Conclusions:**

The simulation tool seems to be very robust in optimising best shape spherical and aspherical lenses based on available data for corneal power and spherical aberration. In all examples the spherical aberrations were completely eliminated or reduced to a negligible amount using individually shaped biconvex aspheric IOLs. An implementation in an industrial manufacturing process of customised aspheric lenses and a clinical study are needed to validate the concept in a clinical setting.

## Background

Various types of intraocular lenses (IOL) are available for replacement of the opaque crystalline lens during cataract surgery. These include spherical lenses (SIOL), aspherical aberration-free / aberration-neutral lenses (ANIOL), aspherical aberration-correcting lenses (ACIOL), toric lenses (TIOL), enhanced depth of field lenses (EdoFIOL) which support an elongated focus for continuous vision from far to intermediate object distances, or multifocal lenses which offer simultaneous vision at 2 or more object distances from far to near distances [4,23].

IOLs are labelled with their paraxial refractive power according to ISO 11979:2024. This refers to the power of a thin equivalent lens located in the secondary (image-sided) principal plane of the IOL. The asphericity of the IOL front or back surface is not considered in the power label [4,23]. The paraxial power can be derived using the central curvature of both surfaces, the central thickness (CT), and the refractive index of the lens (nIOL) and the surrounding media (nA for aqueous humour and nV for the vitreous) [2,9,17]. IOL manufacturers always aim for the thinnest possible IOL design as a trade-off between reducing optical aberrations and enhancing the usability in injectors or folding with a forceps (as thin as possible), and the mechanical stability and a proper support of the IOL haptics (as thick as necessary) [23]. This includes the criterion that the edge thickness (ET) at the optic-haptic junction should have a minimum value, typically ranging between 0.2 and 0.3 mm for the normally used biconvex lens designs. However, based on a minimum ET, the CT is calculated from the diameter of the clear optical zone (OZ, e.g. 6 mm) and the central curvature and asphericity of both IOL surfaces [10]. Most IOLs on the market do not use an equiconvex design (Coddington shape factor CSF = 0), but have larger surface power in the front surface (Pa) and a lower power in the back surface (Pp) (which refers to positive values for the CSF). However, the best aspheric lens design is characterised not only in terms of the CSF and the lens thickness (either CT or ET), but also by the asphericities of both surfaces (Ka and Kp for the front and back surface) [9,10].

There is no algebraic solution for deriving the best IOL shape, as the nominal (labelled) lens power is a function f(Pa, Pp, CT, nIOL, nA, nV), the CT is a function f'(Pa, Pp, Ka, Kp, CT, OZ, nIOL, nA, nV), and the root-mean-squared wavefront error used in this study as a quality metric is determined by the characteristics of the entrance beam (vergence and spherical aberration induced by the cornea), the axial lens position, and the IOL shape.

The purposes of this study were

• to define an optical bench simulation setup environment with a collimated or convergent entrance beam simulating corneal refraction, and an individual biconvex intraocular lens,
• to develop and implement a nonlinear iterative optimisation strategy to derive the best shape aspheric intraocular lens for a preset target corneal spherical aberration, and
• to document the imaging performance of this lens embedded in the optical bench setup environment based on raytracing techniques including wavefront propagation.

## Methods

### Bench setup and raytracing

**Fig. 1:**
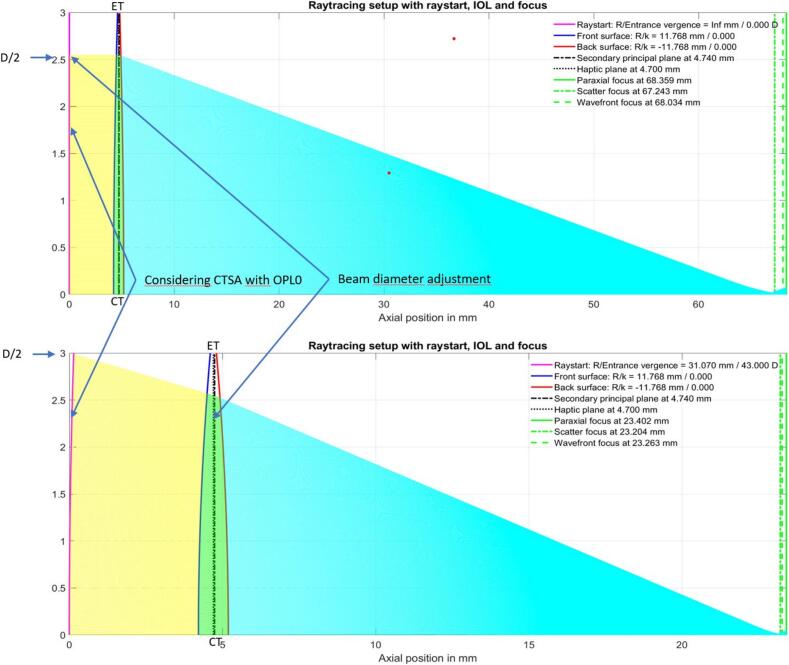
Optical bench setups for defining and optimising for the best shape (a)spheric intraocular lens (IOL): In the upper graph we used a collimated entrance beam with raystart coordinates located in a plane, and in the lower graph we used a convergent beam where the raystart points are located on a circle defined by the entrance vergence VC. To optimise for a correction of a target corneal spherical aberration CTSA (by convention referenced to a standard diameter of 6 mm), we considered an optical path function at baseline OPL0) to be corrected with the IOL. ET and CT refer to the edge and central thickness of the IOL. The diameter of the collimated entrance beam was adjusted to match the corresponding diameter of the convergent beam at the lens plane ELP.

In this simulation study we used a custom 2D raytracing setup to define and optimise a spherical or aspherical IOL and to evaluate the imaging performance. X and Y refer to the axial and lateral direction, and the origin of the coordinate system matches the corneal apex. The IOL was considered in the context of 2 different environments: firstly, with a collimated entrance beam of diameter D with equidistant ray starting point coordinates located at a line with X = 0 and Y = 0 to D/2 (Fig. 1, upper graph), and secondly with a convergent entrance beam having a spherical vergence VC with ray starting point coordinates located on a circle with apex coordinates at X;Y = 0;0 and centre coordinates X;Y = nV/VC;0 and with equal angle increments from 0 to $\text{atan}(\frac{D\cdot VC}{2\cdot nV})$ (Fig. 1, lower graph). To ensure comparability of the results, the ray diameter D of the collimated entrance beam was adjusted to match to the corresponding beam diameter of the convergent beam at the ELP plane. For a systematic wavefront propagation the optical pathlengths (OPL) are tracked for each ray in the bundle by multiplying the geometric distance of sequential ray-surface intersection points by the appropriate refractive index of the media [[11], [12], [13], [14], [15], [16]]. To model a preset corneal target spherical aberration CTSA of the cornea (in a micrometer scale) for the IOL shape optimisation, we added a baseline optical pathlength (OPL0) at the ray startpoints

$$OPL0=\sqrt{5}\cdot CTSA\cdot \left(6\cdot \rho^{4}+6\cdot \rho^{2}+1\right)$$

where ρ refers to the normalised radial distance $\rho =\frac{Y}{D/2}$ for the collimated beam or the normalised arc for the convergent beam. This polynomial term is in accordance to the standardised Zernike polynomial description with the characteristic Z_0_^4^ term for primary spherical aberration [2,8].

The IOL was defined in terms of its nominal lens power IOLP (equivalent power according to ISO 11979), Coddington shape factor CSF, edge thickness ET at a clear optical zone with diameter COZ, refractive index nIOL, assisted with surface radii (Ra and Rp), surface powers (Pa and Pp) and asphericities (Ka and Kp) at the front surface and back surface respectively with

$$\begin{array}{ll} \mathrm{Pa} & =\frac{\mathrm{nIOL}-nA}{\mathrm{Ra}}\\ Pp & =\frac{\mathrm{nV}-nIOL}{\mathrm{Rp}}\\ CSF & =\frac{\left(Rp+Ra\right)}{\left(Rp-Ra\right)}\\ \mathrm{IOLP} & =\mathrm{Pa}+Pp-Pa\cdot Pp\cdot \frac{\mathrm{CT}}{\mathrm{nIOL}}\\ CT & =\mathrm{ET}+\left\{\begin{array}{cc} \frac{{\mathrm{OZ}}^{2}}{8\cdot Ra} & \mathrm{Ka}=-1\\ \frac{\mathrm{Ra}}{1+Ka}\cdot \left(1-\sqrt{1-\left(1+Ka\right)\cdot \frac{{\mathrm{OZ}}^{2}}{\left(4\cdot {\mathrm{Ra}}^{2}\right)}}\right) & \mathrm{Ka}\neq -1 \end{array}\right\}+\\ & \;\;\;+\left\{\begin{array}{cc} \frac{{\mathrm{OZ}}^{2}}{8\cdot Rp} & \mathrm{Kp}=-1\\ \frac{-Rp}{1+Kp}\cdot \left(1-\sqrt{1-\left(1+Kp\right)\cdot \frac{{\mathrm{OZ}}^{2}}{\left(4\cdot {\mathrm{Rp}}^{2}\right)}}\right) & \mathrm{Kp}\neq -1 \end{array}\right\} \end{array}$$

The IOL is assumed to be located with either its secondary (image sided) principal plane (PP2) or its haptic plane (HP) at the effective lens position (ELP), where PP2 and HP, both measured from the front apex of the IOL, are derived from

$$\begin{array}{ccc} \mathrm{HP} & = & 0.5\cdot ET+\left\{\begin{array}{cc} \frac{{\mathrm{OZ}}^{2}}{8\cdot Ra} & \mathrm{Ka}=-1\\ \frac{\mathrm{Ra}}{1+Ka}\cdot \left(1-\sqrt{1-\left(1+Ka\right)\cdot \frac{{\mathrm{OZ}}^{2}}{\left(4\cdot {\mathrm{Ra}}^{2}\right)}}\right) & \mathrm{Ka}\neq -1 \end{array}\right\}\\ PP2 & = & \mathrm{CT}\cdot \left(1-\frac{\mathrm{nV}}{\mathrm{IOLP}}\cdot \frac{\mathrm{nIOL}-nV}{\mathrm{nIOL}\cdot Ra}\right) \end{array}$$

After tracing through the (a)spheric IOL front and back surface, the best focus position was derived by minimising the OPL differences of the ray bundle in terms of minimising the root-mean-squared (RMS) OPL differences amongst the rays in the bundle (RMSWF) [[10], [11], [12], [13], [14], [15], [16]].

### Optimisation strategy for the best shape IOL

**Fig. 2:**
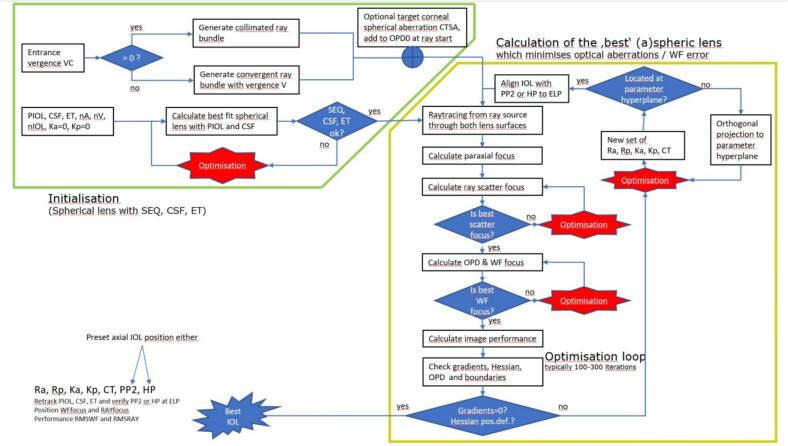
Optimisation strategy: In the initialisation step (green frame) a collimated (corneal vergence CV = 0) or convergent (CV > 0) entrance beam is defined. Corneal target spherical aberration (CTSA) is considered by adding a polynomial function (OPL0) to the optical path length OPL at the raystart point. A best fit biconvex spherical design is predicted from the (labelled) equivalent power (PIOL), edge thickness (ET), and Coddington shape factor (CSF) of the intraocular lens (IOL). After tracing from the raystart points through both IOL surfaces the best wavefront focus (WFfocus) and the best ray scatter focus (RAYfocus) were derived and the appropriate quality metrics root-mean-squared wavefront error (RMSWF) and ray scatter (RMSRAY) were extracted by iteratively adjusting the respective focal plane. After checking the gradients and the Hessian, a new parameter set with IOL front and back radii (Ra and Rp), asphericities (Ka and Kp), and central thickness (CT) was defined. Based on the boundary conditions for PIOL, CSF, and ET the new parameter set was orthogonally projected to the parameter hyperplane, and the projected parameters were used as startpoints for the following iteration. The termination criteria for the optimisation loop were defined as a maximum number of iterations or a step size falling below 1e-10 in a sequence of 5 iterations. From the final parameter set, we extracted Ra, Rp, Ka, Kp, CT, the secondary principal plane (PP2) and the haptic plane (HP) and verified that the resulting PIOL, CSF, and ET matches the preset values and that PP2 or HP was located at the preset effective lens position (ELP). For all optimisations we used the sequential quadratic programming (SQP) technique as this optimisation allows for considering box constraints as well as relational linear and nonlinear boundary conditions.

The optimisation strategy is shown in Fig. 2. For initialisation, either a collimated or convergent entrance beam was defined with N = 301 rays in the hemimeridian (only positive Y values for the startpoints) and the target corneal SA defined by OPL0. A biconvex spherical lens (Ka = Kp = 0) was designed with the target equivalent refractive power PIOL, Coddington shape factor CSF, and edge thickness ET within the clear optical zone OZ (upper left part of the graph). In the optimisation sequence (lower right part of the graph), we shifted the lens to match either its PP2 or HP to the preset ELP. We then traced through the 2 spherical or aspherical IOL surfaces searching for the best wavefront focus (X = WFfocus) in terms of minimising the RMSWF, and for the best ray scatter focus (RAYfocus) in terms of minimising the RMS ray scatter (RMSRAY) of ray intersection with the focal plane located at X = RAYfocus. Next, the gradients and the Hessian of the quality metric RMSWF with respect to Ra, Rp, Ka, Kp, and CT were evaluated to extract the increment step for the following iteration [1,3,7,20]. While maintaining the nonlinear relational boundary conditions for CSF, PIOL and ET, the new parameter set in the parameter space was derived by orthogonal projection to the parameter hyperplane described by the boundary conditions [24,27,28]. This orthogonal projection point located at the hyperplane was used as the starting point for the next iteration. The iteration cycle terminated when a predefined number of iterations (1000 iterations) was reached or when the step size for 5 iterations in a sequence fell below 1⋅10^−10^. The SQP (sequential quadratic programming) algorithm was used for all optimisations [3,7]. When exiting the iteration loop, the characteristic IOL parameters Ra, Rp, Ka, Kp and CT were documented along with RMSWF, WFfocus, RMSRAY, RAYfocus. The input parameters IOLP, ET, were also tracked and compared with the corresponding preset values for verification together with the match of PP2 or HP with the ELP [[9], [10], [11], [12], [13], [14], [15], [16]].

The program code including raytracing and optimisation was implemented in Matlab (The Math Works, version 2024a) using class libraries. Some working examples are shown in the Results section proving the applicability of this calculation strategy to predict the design of best shape lenses.

## Results

For all simulation examples we used the following preset values without loss of generality: nA = nV = 1.336, nIOL = 1.46, CV = 43 dioptres (D) for the convergent entrance beam, ET = 0.2 mm and OZ = 6 mm for the lens design, a ray bundle diameter of D = 6 mm considered at the corneal plane for the convergent beam or reduced to 5.09 mm (dependent on the CV and ELP) for the collimated beam, and an ELP = 4.7 mm.

**Fig. 3:**
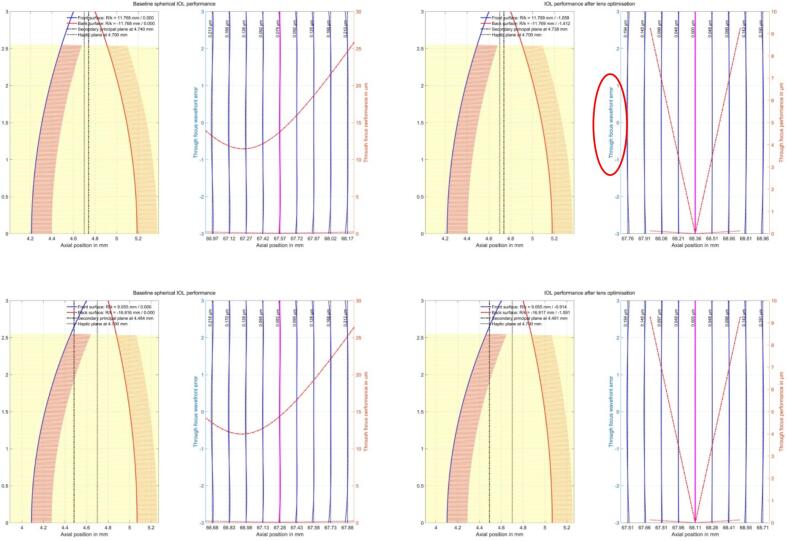
Simulation example 1: Optimising the lens performance in a bench environment with a collimated entrance beam (beam diameter adjusted to match to the beam diameter of the convergent beam at the lens position ELP shown in examples 2-4). The lens is assumed to be located with its haptic plane HP at the predicted effective lens position ELP (4.7 mm). The left graphs show the situation with the best shape spherical lens, and the right graphs with the best shape aspheric lens. In the upper graphs we used an equiconvex lens design with Coddington shape factor CSF = 0, and in the lower graphs we used an asymmetric design with a CSF = 0.3. The secondary principal plane PP2 is shown in the graphs (slightly posterior to the haptic plane for the equiconvex and systematically to the anterior of the haptic plane with the asymmetric lens). Optimisation was performed in terms of minimising the root mean squared (RMS) wavefront error, and the performance curves for the RMS wavefront error (dotted red line) and the RMS ray scatter (dash-dotted red line) are overlaid on the wavefront plots. The right hand plot in each of the four subfigures shows the wavefronts progressing from left to right. In each plot, the blue lines on the left show the wavefronts approaching the wavefront focal plane, the best wavefront focus is marked with the magenta line, and the blue lines on the right show the wavefronts behind the focal plane. The values in black at the top of the plot indicate the RMS wavefront error at each position. With the best shape spherical lenses the location of the ray scatter focus RAYfocus is located more towards the anterior as compared to the wavefront focus WFfocus. After lens optimisation (right hand two subfigures) the ray scatter focus and the wavefront focus coincide. Please note that the simulation setup with a collimated beam according to ISO 11979 is not suitable for retrieving a realistic focus position (here around 68 mm) or image performance (rms wavefront error or ray scatter).

In example 1 we defined the IOL located with its HP at the ELP, and used a collimated entrance beam and an aberration free cornea (CTSA = 0). The Coddington shape factor CSF was varied (0.0 referring to an equiconvex lens and 0.3 to a more curved front surface) to show the impact of the general IOL shape on the geometry of the best fit spherical SIOL and aberration neutral ANIOL. Fig. 3 presents the corresponding results with the best SIOL shown in the left graphs and the best shape ANIOL in the right graphs. The upper row refers to the equiconvex lens design (CDF = 0) with the PP2 located shortly behind the HP and the lower row refers to the more curved asymmetrical lens design (CSF = 0.3) where the PP2 is systematically shifted to the anterior. In the unrealistic simulation scenario of a collimated entrance beam according to ISO 11979 the WFfocus of the equiconvex lens is located at 67.57 mm/at 68.36 mm with the SIOL/ANIOL, and at 67.28 mm/68.11 mm for the asymmetric lens. For the equiconvex/asymmetrical SIOL the RMSWF is 0.082 µm/0.078 µm and the RAYfocus is systematically shifted to the anterior compared to the WFfocus as shown in the performance curves. However, after optimising for the best shape ANIOL, both the equiconvex and the asymmetrical IOL showed excellent performance with negligible RMSWF and the RAYfocus is properly aligned with the WFfocus.

**Fig. 4:**
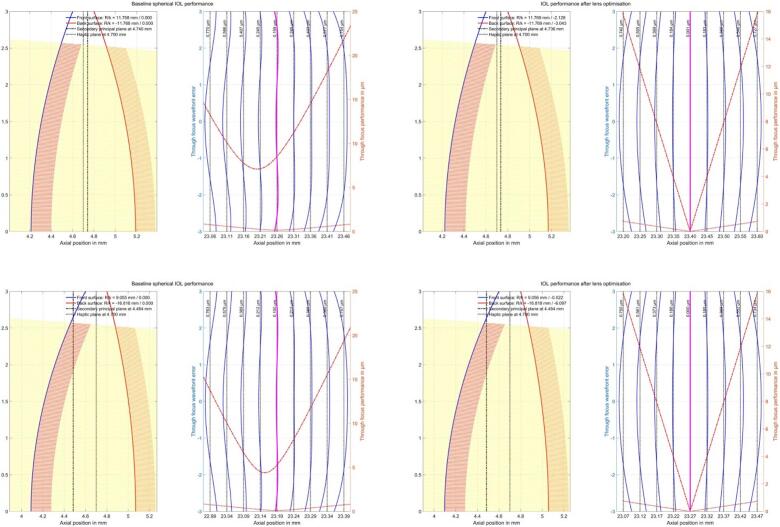
Simulation example 2: Optimising the lens performance in a bench environment with a convergent entrance beam (vergence 43 D to simulate the refracting behaviour of a normal cornea). The lens is assumed to be located with its haptic plane HP at the predicted effective lens position ELP (4.7 mm). The left graphs show the situation with the best shape spherical lens, and the right graphs with the best shape aspheric lens. In the upper graphs we used an equiconvex lens design with Coddington shape factor CSF = 0, and in the lower graphs we used an asymmetric design with a CSF = 0.3. The secondary principal plane PP2 is shown in the graphs (slightly posterior to the haptic plane for the equiconvex and systematically anterior to the haptic plane with the asymmetric lens). Optimisation was performed in terms of minimising the root mean squared (RMS) wavefront error, and the performance curves for the RMS wavefront error (dotted red line) and the RMS ray scatter (dash-dotted red line) are overlaid on the wavefront plots. The right hand plot in each of the four subfigures shows the wavefronts progressing from left to right. In each plot, the blue lines on the left show the wavefronts approaching the wavefront focal plane, the best wavefront focus is marked with the magenta line, and the blue lines on the right show the wavefronts behind the focal plane. The values in black at the top of the plot indicate the RMS wavefront error at each position. With the best shape spherical lenses the location of the ray scatter focus RAYfocus is located more towards the anterior as compared to the wavefront focus WFfocus. After lens optimisation (right hand two subfigures) the ray scatter focus and the wavefront focus coincide.

In example 2 we used a convergent entrance beam with CV = 43 D instead of a collimated beam. The IOL was again located with its HP at the ELP, with an aberration free cornea (CTSA = 0), and the Coddington shape factor CSF was varied (0.0 referring to an equiconvex lens and 0.3 to a more curved front surface) to show the impact of the general IOL shape on the geometry of the best fit spherical SIOL and aberration neutral ANIOL. Fig. 4 presents the corresponding results with the best SIOL shown in the left graphs and the best shape ANIOL in the right graphs. In the realistic simulation scenario with a convergent entrance beam (instead of the collimated beam defined in ISO 11979) the WFfocus of the equiconvex lens is located at 23.26 mm/at 23.19 mm with the SIOL/ANIOL, and at 23.40 mm/23.27 mm for the asymmetric lens. For the equiconvex/asymmetrical SIOL the RMSWF (0.159 µm/0.100 µm) is much larger compared to example1 and the RAYfocus is systematically displaced to the anterior compared to the WFfocus as shown in the performance curves. However, after optimising for the best shape ANIOL, both the equiconvex and the asymmetrical IOL showed excellent performance with negligible RMSWF (0.001 µm and 0.000 µm) and with the RAYfocus properly aligned with the WFfocus.

**Fig. 5:**
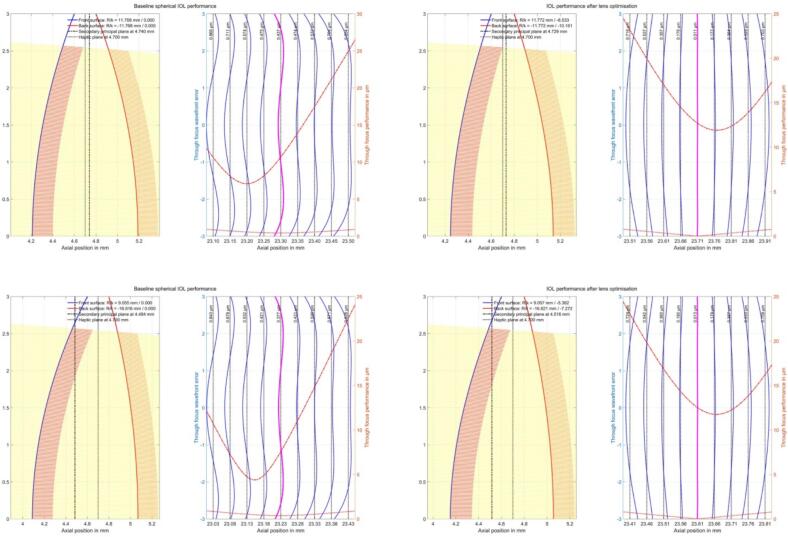
Simulation example 3: Optimising the lens performance in a bench environment with a convergent entrance beam (vergence 43 D to simulate the refracting behaviour of a normal cornea). The lens is assumed to be located with its haptic plane HP at the predicted effective lens position ELP (4.7 mm). In contrast to example 2 we considered a corneal spherical aberration of CTSA = 0.27 (6 mm zone, according to the Liou Brennan schematic model eye) to be corrected by the lens. The left graphs show the situation with the best shape spherical lens, and the right graphs with the best shape aspheric lens. In the upper graphs we used an equiconvex lens design with Coddington shape factor CSF = 0, and in the lower graphs we used an asymmetric design with a CSF = 0.3. The secondary principal plane PP2 is shown in the graphs (slightly posterior to the haptic plane for the equiconvex and systematically anterior to the haptic plane with the asymmetric lens). Optimisation was performed in terms of minimising the root mean squared (RMS) wavefront error, and the performance curves for the RMS wavefront error (dotted red line) and the RMS ray scatter (dash-dotted red line) are overlaid on the wavefront plots. The right hand plot in each of the four subfigures shows the wavefronts progressing from left to right. In each plot, the blue lines on the left show the wavefronts approaching the wavefront focal plane, the best wavefront focus is marked with the magenta line, and the blue lines on the right show the wavefronts behind the focal plane. The values in black at the top of the plot indicate the RMS wavefront error at each position. With the best shape spherical lenses the location of the ray scatter focus RAYfocus is located more towards the anterior as compared to the wavefront focus WFfocus. Please note that with the best shape spherical lenses there is a large amount of spherical aberration left in the wavefront which includes corneal and lens spherical aberration. With the best shape aspheric lens the RMS wavefront error could be mostly eliminated.

In example 3 we wanted to create an aberration correcting aspheric ACIOL which would compensate the nominal CTSA value of 0.27 µm within a 6 mm optical zone of the cornea as documented in the Liou-Brennan schematic model eye [2,17,22]. We again used a convergent entrance beam with CV = 43 D, with the IOL HP located at the ELP, and the Coddington shape factor CSF varied (0.0 referring to an equiconvex lens and 0.3 to a more curved front surface) to show the impact of the general IOL shape on the geometry of the best fit spherical SIOL and aberration neutral ACIOL. Fig. 5 presents the corresponding results with the best SIOL shown in the left graphs and the best shape ANIOL in the right graphs. With the convergent entrance beam the WFfocus of the equiconvex lens is located at 23.30 mm/at 23.25 mm with the SIOL/ANIOL, and at 23.71 mm/23.66 mm for the asymmetric lens. For the equiconvex/asymmetrical SIOL the RMSWF is 0.457 µm/0.377 µm and the RAYfocus is systematically shifted to the anterior compared to the WFfocus as shown in the performance curves. However, after optimising for the best shape ANIOL both the equiconvex and the asymmetrical IOL showed very good performance with a small RMSWF (0.011 µm and 0.013 µm) and the RAYfocus located slightly to the posterior of the WFfocus. This example shows that even with a large SA value derived with the spherical equiconvex or asymmetrical SIOL after optimisation, the residual spherical aberration could be significantly reduced to around 11 or 13 nm, but also that a full aberration correction seems to be unrealistic with such an ACIOL with individually shaped aspheric front and back surface. It has to be noted that with the equiconvex ACIOL much larger (negative) values for Ka/Kp = −6.533/−10.161 are required to correct for the spherical aberration as compared to the corresponding values for the asymmetric ACIOL with Ka/Kp = −5.362/−7.272.

**Fig. 6:**
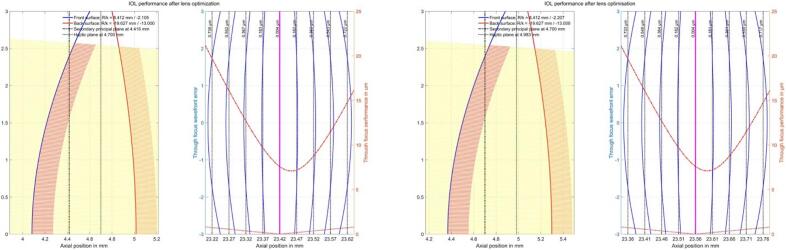
Simulation example 4: Optimising the lens performance in a bench environment with a convergent entrance beam (vergence 43 D to simulate the refracting behaviour of a normal cornea). The optimised aspheric lens is assumed to be located with its haptic plane HP (left graph) or its secondary principal plane PP2 (right graph) at the predicted effective lens position ELP (4.7 mm). We considered a corneal spherical aberration of CTSA = 0.16 (6 mm zone) to be corrected by the lens. A Coddington shape factor CSF = 0.4 was used to underline the differences in the resulting focus position. The secondary principal plane PP2 is shown in the graphs (slightly posterior to the haptic plane for the equiconvex and systematically anterior to the haptic plane with the asymmetric lens). Optimisation was performed in terms of minimising the root mean squared (RMS) wavefront error, and the performance curves for the RMS wavefront error (dotted red line) and the RMS ray scatter (dash-dotted red line) are overlaid on the wavefront plots. The right hand plot in each of the four subfigures shows the wavefronts progressing from left to right. In each plot, the blue lines on the left show the wavefronts approaching the wavefront focal plane, the best wavefront focus is marked with the magenta line, and the blue lines on the right show the wavefronts behind the focal plane. The values in black at the top of the plot indicate the RMS wavefront error at each position. Please note that with adjusting the HP or PP2 to the ELP the WFfocus differs by 0.19 mm.

In example 4 we wanted to create an aberration correcting aspheric ACIOL which would compensate the nominal CTSA value of 0.16 µm within a 6 mm optical zone of the cornea. The Coddington shape factor CSF was defined to be 0.4 (asymmetric front-dominant IOL) and we again used a convergent entrance beam with CV = 43 D. The IOL was located with either with HP or its PP2 at the ELP to show the impact of the philosophy of the IOL calculation formula specific prediction model for the ELP (prediction of either the HP or the PP2) on the focus position and on the image performance of the best fit aberration neutral ACIOL. Fig. 6 presents the corresponding results with the best ACIOL located with its HP at the ELP (left graph), and the best shape ACIOL located with its PP2 at the ELP (right graph). With the HP at the ELP the PP2 is located at 4.42 mm (WFfocus at 23.42 mm), and with the PP2 at ELP the HP is located at 4.98 mm (WFfocus at 23.56 mm). As noted in the legends the radii (Ra and Rp) and asphericities (Ka and Kp) are slightly changed to reach the goal of correcting the CTSA. Both scenarios show an excellent image performance with a RMSWF of 0.004 µm.

## Discussion

Recent years have seen much discussion about the general shape of intraocular lenses in terms of symmetric or asymmetric designs and spherical or aspherical surfaces [[4], [5], [6],^18^,^19^,^25^,^26^]. SIOLs are well known to show excellent tolerance for decentration or tilt and are mostly preferred in cases where a poor centration of the IOL might be expected from the preoperative situation of the patient [21,22,26]. However, aspherical lenses which are capable of maintaining the corneal spherical aberration (ANIOL) or even correcting the corneal spherical aberration to some extent yield a significantly better image performance compared to SIOLs if properly centred, especially with a larger pupil [4,9,23]. There is however no consensus in the literature about the precise definition of the terms ‘aberration-neutral’ or ‘aberration correcting’ [4,23]. Most IOL manufacturers design their IOL models using commercial raytracing software tools such as ZEMAX, with a simulation environment based on a classical schematic model eye. For example, with an ANIOL design we could use either a collimated or a convergent entrance beam. These two scenarios yield systematically different results: if we optimise our ANIOL in a collimated beam we require much less (negative) asphericity in the lens (example 1, Fig. 3 right graphs) compared to optimising the ANIOL in a convergent beam (example 2, Fig. 4 right graphs). Consequently, an ANIOL optimised in a collimated beam will act as an ANIOL in a convergent beam (located behind a cornea) but will induce some positive spherical aberration, and vice-versa. An ANIOL optimised in a convergent beam will act as a ACIOL with induction of negative spherical aberration [10].

Since most modern corneal or anterior segment topographers or tomographers provide data on corneal spherical aberration in addition to corneal power we decided to use the corneal vergence CV (referring to the refraction of an ideal cornea) and a corneal target spherical aberration CTSA in our simulation study. By convention, spherical aberration of the cornea is referenced to a central 6 mm zone, and we adapted our simulation to directly use this spherical aberration value as CTSA for optimising for the best shape spheric or aspheric lens. However, when using CV and CTSA, the lack of corneal shape data means that the classical way of raytracing through the cornea and the lens cannot be used any more [8,9]. To counter this, we switched to a hybrid raytracing optimisation which uses raystart coordinates located on a line (for the collimated beam) or on a circle (for the convergent beam) to trace the ray bundle through the IOL. A wavefront optimisation was then used to find the best WFfocus used to optimise the IOL design. With this strategy we are able to add a spherical aberration (or in principle any other wavefront aberration profile for the cornea) in terms of adding a baseline optical path length profile OPL0 at the raystart points to the OPL [15].

Since lens manufacturers always aim for lenses with large optical zones but small volumes to support minimal invasive cataract surgery (MICS) with a small corneal incision, the mechanical stability of the typically used biconvex IOL is determined by its edge thickness ET which supports the haptic junctions of the lens. We therefore developed a concept to prioritise the edge thickness by using a preset value for the ET instead of the CT. However, the nominal lens power PIOL is derived from the simple Gullstrand formula for thick lenses which involves CT rather than ET. This increases the complexity of the optimisation, since the conversion of ET to CT is rather complex and involves Ra, Rp, Ka, Kp together with the refractive indices nA, nIOL and nV. The definition of nominal lens power according to ISO 11979 as paraxial equivalent power implies that the thin replacement lens is located at the PP2. However, as PP2 depends on the CSF of the lens and the PP2 is typically not disclosed by the IOL manufacturer and typically varies over the entire delivery range, it is rather difficult to develop a general prediction concept for the axial lens position in terms of PP2 with an IOL prediction formula. We therefore developed a strategy to derive the HP from the lens shape data to be used in our optimisation. This HP allows a much easier handling, and coincides with the lens plane in any optical bench measurement setup [26]. However, the determination of the HP plane is rather complex as it includes CSF, Ka, Kp, OZ, and ET in addition to Ra and Rp. Nevertheless, we feel that the HP much better represents the reference plane of the lens in terms of the lens capsule equator which could be easier predicted with a common IOL power formula. Example 4 shows the difference between both axial positioning concepts. Especially with asymmetric lens designs (CSF ≠ 0), the HP differs systematically from the PP2. Consequently, using a more realistic prediction model for the HP with an IOL calculation formula, the PP2 plane (which is referenced to the IOLP according to ISO 11979) could differ significantly from the HP making prediction of a proper lens power or postoperative refraction of the patient after cataract surgery unreliable.

**Fig. 7:**
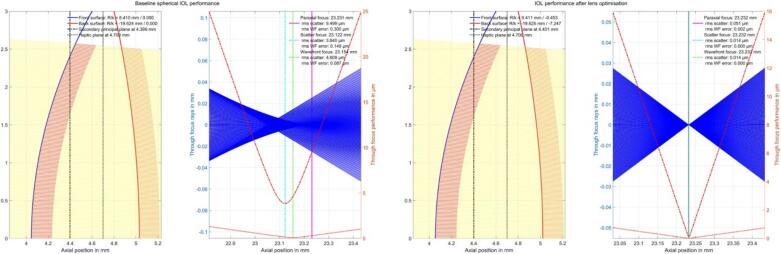
Example of a classical raytracing setup with optimising the lens performance in a bench environment with a convergent entrance beam (vergence 43 D to simulate the refracting behaviour of a normal cornea without considering corneal spherical aberration). The best shape spherical lens (left graph) shows a large amount of spherical aberration (peripheral rays are more refracted compared to paraxial rays), and the wavefront and rayscatter focus are both located to the anterior of paraxial focus. After optimising for the best shape aspheric lens (right graph) the rayscatter focus and the wavefront focus are aligned at 23.23 mm. We considered a Coddington shape factor CSF = 0.4. The secondary principal plane PP2 is shown in the graphs (slightly posterior to the haptic plane for the equiconvex and systematically anterior to the haptic plane with the asymmetric lens). Optimisation was performed in terms of minimising the root mean squared (RMS) wavefront error, and the performance curves for the RMS wavefront error and the RMS ray scatter are overlaid on the wavefront plots (red lines). At the focus the RMS ray scatter/RMS wavefront errors are 0.014 µm/0.000 µm respectively.

Fig. 7 shows the classical raytracing approach where the image performance is optimised by minimising the ray scatter RMSRAY at the focal plane. However, this concept is restricted to using either corneal topographic or tomographic measurement data for raytracing [11,15], or alternatively to using a collimated or convergent entrance beam without any correction of corneal spherical aberration CTSA by the lens. This classical approach does not yield proper results when using the CV in combination with any CTSA ≠ 0 because the baseline optical path length OPL0 does not affect the refraction of the ray bundle in classical raytracing. However, when corneal topography or tomography is available these data already include corneal spherical aberration and classical raytracing is expected to give proper results. In the left graph showing the ray image for the best shape SIOL (CSF = 0.4) lens the spherical aberration is clearly visible (marginal rays are systematically more refracted compared to paraxial rays), and the RAYfocus and WFfocus both are systematically located to the anterior of the paraxial focus. The RMSRAY is 9.50 µm and the RMSWF is 0.087 µm. After lens optimisation, the resulting ABIOL shows an excellent performance, and the RAYfocus and the WFfocus are properly aligned (both at 23.23 mm). The resulting RMSRAY and RMSWF yield 0.014 µm / 0.00 µm respectively.

Merging all of the formulae shown in the Methods section together and searching for the best shape spheric/aspheric IOL which gives the best imaging when located with its PP2 or HP at the preset ELP cannot be solved algebraically. We therefore used a numerical optimisation strategy which searches iteratively for the best solution for the lens [1,3,7]. Most of the optimisation tools used by scientists are based on Gauss-Newton, gradient descent or direct line search strategies, but most of these are restricted to implement box constrains for the parameter space [1]. However, in in our case when optimising our 5 parameters Ra, Rp, Ka, Kp, and CT simultaneously, we have to consider several nonlinear relational boundary conditions [3,20,24,27,28]. For example, the IOLP derived from Ra, Rp CT and the refractive indices should match our preset value, the CSF derived from Ra and Rp should match our preset value, and the ET derived from Ra, Rp, Ka, Kp, CT and the refractive indices should match the preset value. To manage these complex relational constrains together with simple box constrains, we decided to use the SQP algorithm for optimisation [3]. Our examples, with additional tests outside the scope of this paper, prove that this SQP algorithm showed excellent robustness and did not fail to optimise proper lens designs in any case.

However, our study has some **limitations**: 1) we used 2D raytracing instead of 3D raytracing. 2D raytracing assumes a coaxially centred optical system, and is essentially limited to rotationally symmetric corneas and IOLs and any type of rotationally symmetric corneal aberrations. This was intended in this study to find the best shape spheric/aspheric rotationally symmetric IOL. The concept could be generalised to 3D raytracing in order to include asymmetric corneal aberrations or IOL designs which are not rotationally symmetric [8,11,13]. However, we feel that the optimisation strategy that we presented would work properly for both 2D and 3D raytracing. 2) In this study we assumed that the lens power is labelled correctly by the IOL manufacturer according to ISO 11979. However, as the IOL design has to be taken into account when defining the PP2 for lens labelling, the IOLP cannot be measured or approved with any optical bench to derive the nominal optical power without knowledge of the design. Therefore switching to the HP, which could provide a good reference for both optical simulations and optical bench measurements, could be a serious option.3) The new calculation concept shown in this paper has not yet been implemented in an industrial manufacturing process, and we do not have clinical results to show the superiority of our calculation compared to state of the art lenses designed with schematic model eyes. However, we argue that our calculation strategy which could easily be used to calculate individual rotationally symmetric lenses based on biometric and topographical patient data could be a new field for innovative IOL manufacturers which aim for better vision with customised implants in cataract surgery.

**In conclusion**, in this paper we developed and implemented a novel concept for individually optimising spherical or aspherical IOLs based on the vergence and spherical aberration of the cornea and the predicted axial lens position. The lens optimisation included a setup with both a collimated and a convergent entrance beam, and the lens positioned with its secondary principal plane or its haptic plane at the predicted axial position to keep full flexibility for the underlying lens power calculation formulae. An industrial implementation in a lens manufacturing process and clinical studies are required to prove the applicability in a clinical setup for customised lens implants in cataract surgery.

**Table:** 

**List of symbols and abbreviations**	
ACIOL	Aberration correcting lens, a lens which is designed to correct (fully or to some extent) the corneal spherical aberration
ANIOL	Aberration neutral lens, a lens which is designed not to modify the spherical aberration of the eye
CSF	Coddington shape factor derived from central front and back surface curvature
CT	Central lens thickness on mm
CTSA	Target value for the spherical aberration correction in µm
CV	Vergence of the entrance beam which specifies the refracting power of the cornea
D	Diameter of the input ray bundle used for raytracing
ELP	Effective lens position considered from corneal apex (origin of the coordinate system) to either the haptic plane or the secondary principal plane of the lens in mm
ET	Thickness of the lens optical edge in mm
HP	Lens haptic plane which refers to the plane which bisects the optical edge of the lens
IOL	Intraocular lens
ISO 11979	Relevant DIN-ISO Standard which specifies lens types and parameters
Ka	Lens front surface asphericity
Kp	Lens back surface asphericity
MICS	Minimal invasive cataract surgery
OPL	Optical path length
OPL0	Target value for the spherical aberration correction transformed into a wavefront profile at the raystart plane
OZ	Clear optical zone of the lens in mm (where the edge thickness ET is considered)
PIOL	Lens nominal equivalent power in D (to be labelled on the package)
PP1	Object-side principal plane of the lens
PP2	Image-side principal plane of the lens
Ra	Lens front surface radius of curvature in mm
RAYfocus	Best focus position in terms of minimal root-mean-squared rayscatter in the image plane in mm
RMS	root-mean-squared
RMSRAY	Root mean squared ray scatter at focal plane
RMSWF	Root mean squared wavefront error in terms of optical pathlength differences in µm
Rp	Lens back surface radius of curvature in mm
SIOL	Spherical lens, a lens with spherical front and back surfaces
SQP	Sequential Quadratic Programming nonlinear iterative optimisation algorithm used for all parametric univariate and multivariate optimisations
WFfocus	Best focus position in terms of minimal root-mean-squared optical path length differences from object plane to image plane in mm
ZEMAX	Example of a commercial optics design software

## CRediT authorship contribution statement

**Achim Langenbucher:** Writing – original draft, Validation, Software, Methodology, Investigation, Formal analysis, Data curation, Conceptualization. **Jascha Wendelstein:** Supervision, Formal analysis, Data curation. **Alan Cayless:** Writing – original draft, Methodology, Investigation. **Peter Hoffmann:** Validation, Formal analysis. **Nóra Szentmáry:** Writing – original draft, Validation, Project administration, Methodology.

## Declaration of competing interest

The authors declare the following financial interests/personal relationships which may be considered as potential competing interests: AL: Speakers fees from Bausch & Lomb and Johnson & Johnson unrelated to the presented material; NS & AC: None; PH: Speakers fees from Heidelberg Engineering, Hoya Surgical and Johnson & Johnson unrelated to the presented material; JW: speakers fees from Hoya Surgical, Johnson & Johnson and Carl-Zeiss-Meditec unrelated to the presented material. The author is an Editorial Board Member/Editor-in-Chief/Associate Editor/Guest Editor for this journal and was not involved in the editorial review or the decision to publish this article.
